# The Role of Non-Coding RNAs in Liver Disease, Injury, and Regeneration

**DOI:** 10.3390/cells12030359

**Published:** 2023-01-18

**Authors:** Melissa M. Rowe, Klaus H. Kaestner

**Affiliations:** Department of Genetics and Center for Molecular Studies in Digestive and Liver Diseases, University of Pennsylvania, Philadelphia, PA 19014, USA

**Keywords:** non-coding RNA, microRNA, long non-coding RNA, circular RNA, liver disease, liver regeneration

## Abstract

Non-coding RNAs (ncRNAs) have diverse functions in health and pathology in many tissues, including the liver. This review highlights important microRNAs (miRs), long non-coding RNAs (lncRNAs), and circular RNAs (circRNAs) in liver disease and regeneration. Greater attention is given to more prevalent and well characterized RNAs, including: miR-122, miR-21, the let-7 family of miRs, miR-451a, miR-144, and MALAT1.

## 1. Introduction: Liver Disease

Chronic liver disease is a major health burden worldwide, causing an estimated 1.32 million deaths globally in 2017 [1]. The majority of these deaths can be attributed to cirrhosis, i.e., advanced scarring of the liver, which is considered the last stage of chronic liver disease [2]. The majority of liver cirrhosis cases are due to hepatitis B virus (HBV) or hepatitis C virus (HCV) infection, with over half of global cirrhosis deaths in 2017 being attributed to viral hepatitis [1]. Both HBV and HCV can cause liver injury through their own activity, such as creating genetic alterations in the host cell and prioritizing cellular pathways that create viral proteins, and via the host immune response. The latter mechanism of injury is largely modulated by cytotoxic T lymphocytes, although cytokine release and Kupffer cell activity can both promote liver injury in viral hepatitis [3,4]. The second leading cause of liver cirrhosis is alcoholic liver disease, caused by the long-term overconsumption of excessive amounts of ethanol [1]. Non-alcoholic fatty liver disease (NAFLD) and non-alcoholic steatohepatitis (NASH) are also significant contributors to global chronic liver disease [1]. NAFLD is typically defined as fat accumulation in over 5% of hepatocytes without another apparent cause (such as alcohol consumption or viral infection) [5]. NASH is diagnosed when hepatic fat accumulation is paired with inflammation and damage to the liver, which can include fibrosis and cirrhosis [5,6]. An estimated 30–40% of NAFLD cases will progress to NASH [5].

Hepatocellular carcinoma (HCC) also contributes significantly to the burden of liver disease worldwide. Liver cancer is the second leading cause of cancer deaths globally, with HCC representing around 80% of liver cancer diagnoses [7]. The current model of HCC is that oncogenic transformation occurs in one or more mature hepatocytes which then rapidly proliferate to form tumors. However, there are ongoing discussions in the field regarding whether HCC can arise when another cell type within the liver differentiates or re-differentiates to become a cancerous hepatocyte [8].

The largely postmitotic liver retains a remarkable regenerative ability throughout life. It can weather the loss of up to two-thirds of its mass and still regenerate back to its original size [9]. For this regenerative process, the liver does not rely on dedicated stem or progenitor cells such as those present in the intestine; rather, quiescent hepatocytes reenter the cell cycle en masse to restore liver tissue [10]. Given this impressive regenerative ability and the toll liver diseases take across the globe, investigating the mechanisms of liver injury and regeneration promises to shed light on a variety of pressing medical questions. Thus, our understanding of cancer, aging, and ex vivo growth of tissues for transplantation will be significantly enriched by the molecular analysis of the mechanisms regulating hepatic regrowth.

## 2. The Major Types of Non-Coding RNAs

One topic of particular interest in liver biology is epigenetic regulation by non-coding RNAs. Non-coding RNAs (ncRNAs) encompass all RNAs that are not translated into proteins. They are classified by features such as length and structure, and include microRNAs (miRs), long non-coding RNAs (lncRNAs), and circular RNAs (circRNAs). These different classes of ncRNAs participate in complex interaction networks with each other, mRNAs, and various signaling pathways to enact changes on cells, surrounding tissue, and whole organ systems.

**Figure 1:**
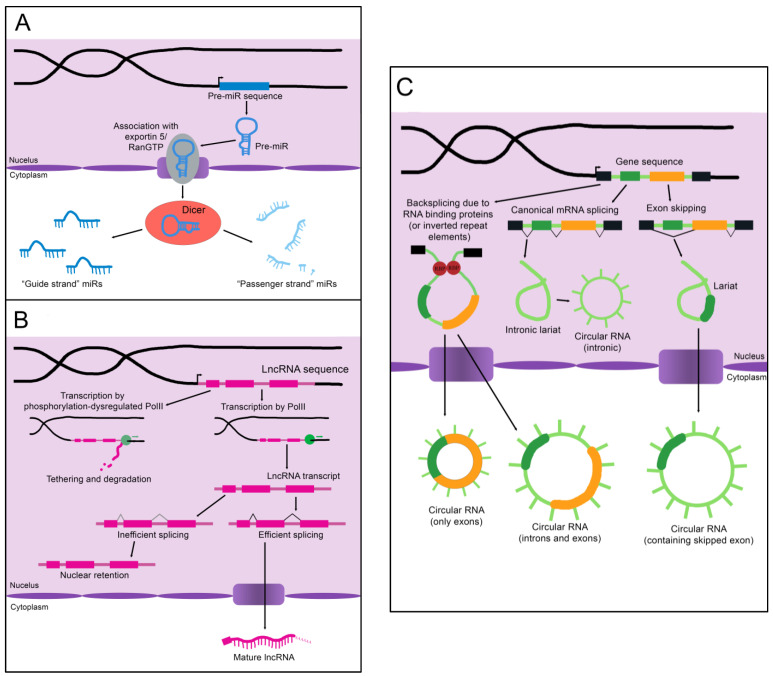
The biogenesis of non-coding RNAs. (**A**) An immature miR transcript is transcribed and exported into the cytoplasm by Exportin 5 bound to Ran-GTP. The “guide” and “passenger” strands are created when the Dicer nuclease splits and processes these precursors. (**B**) The sequence of a lncRNA is transcribed by RNA polymerase II (PolII) and either spliced to its mature form or inefficiently spliced and retained in the nucleus. Alternatively, the sequence is transcribed by phosphorylation-dysregulated PolII, remains tethered to the chromatin, and either accumulates over time or is degraded. (**C**) CircRNAs are created through back-splicing mediated by RNA binding proteins, inverted repeat elements, or lariats created during mRNA splicing. The mature circRNA may contain only exons, only introns, or a combination of both. RBP: RNA-binding protein.

MicroRNAs (miRs) are the smallest of the non-coding RNAs, with an average size of 22 nucleotides [11]. A primary miR (pri-miR), which may be kilobases long, is transcribed from its genomic coding region [12]. The Microprocessor complex trims and processes the pri-miR to create a 60–90 nucleotide precursor miR (pre-miR) [12]. The pre-miR often exists as a double-stranded hairpin loop and is exported into the cytoplasm by Exportin 5 bound to Ran-GTP (see Figure 1A) [13]. Two mature miRs are created when the Dicer nuclease splits and processes the strands [13]. These mature miRs are then referred to as the “5p” or “3p” species, coming from the 5′ and 3′ ends of the pre-miR transcript, respectively [14]. However, not all mature miRs are created equal. Often, either the 5p or 3p strand is expressed more highly and has a stronger effect on epigenetic gene regulation (and is thus referred to as the “guide strand”), while the other “passenger strand” is frequently (but not always) degraded [13]. Advancing technology has recently established that stably expressed passenger strands are more common than previously thought, and further studies are warranted to uncover unknown functions of these miR species [14]. Some studies refer only to the name of the miR without defining the 5p or 3p species, while others specify which molecule is assigned a given biological function. We will attempt to be as clear as possible in specifying the mature miR being described; however, it is sometimes necessary to list certain functions and disease associations without specifying the strand if it is unclear from the examined literature whether one or both strands are functionally active.

**Figure 2:**
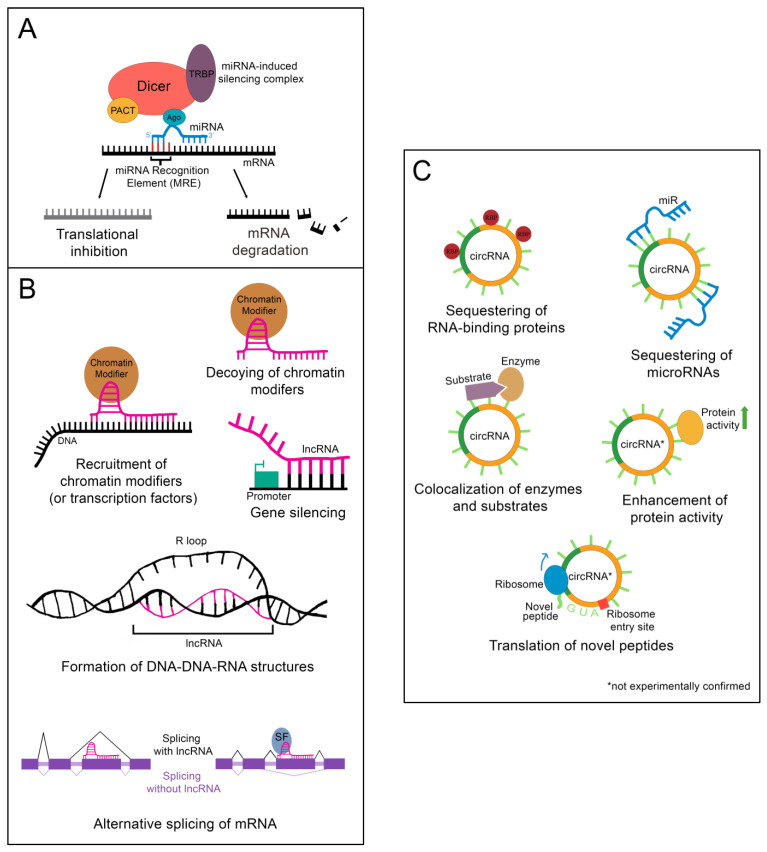
Epigenetic regulation by ncRNAs. (**A**) The microRNA-induced silencing complex downregulates mRNAs through translational inhibition or degradation. TRBP: transactivation response element RNA-binding protein; PACT: protein kinase RNA activator; Ago: Argonaute (**B**) lncRNAs alter gene expression by binding DNA, binding mRNA, or by sequestering chromatin modifiers. DNA binding by lncRNAs can result in chromatin modifier or transcription factor recruitment, gene silencing, or the formation of DNA-DNA-RNA structures. Binding of a lncRNA to mRNA often leads to alternative splicing of the mRNA. For instance, the presence of a lncRNA on an exon can lead to that exon being skipped. Alternatively, recruitment of splicing factors by a bound lncRNA can lead to the retention of an exon that would otherwise be skipped. SF: splicing factor (**C**) CircRNAs function as sponges for RNA-binding proteins or microRNAs. Some can also colocalize enzymes with their substrates. A few are proposed to enhance protein activity or be translated into novel peptides, although these functions have yet to be experimentally validated. RBP: RNA-binding protein.

Following processing, the mature miR associates with Argonaute protein (Ago), transactivation response element RNA-binding protein (TRBP), protein kinase RNA activator (PACT), and the still-bound Dicer to create the microRNA-induced silencing complex (miRISC), which carries out the epigenetic suppression of target mRNAs (Figure 2A) [13]. The identification of target mRNAs is not trivial and currently cannot be done reliably using only computational tools. This is because only 6–8 consecutive nucleotides in the miR’s 5′ “core” sequence are required to bind the microRNA recognition element (MRE) in a target mRNA [13]. In addition, imperfect base pairing can occur between the core sequence and MREs [13]. MiR to MRE interactions are also able to make G:U pairs, providing additional flexibility in target binding [13]. Due to the variable nature of this process, a given miR can have hundreds of potential mRNA targets [13]. Fortunately, biochemical methods such as CLIP-Seq (cross-linking immunoprecipitation followed by high throughput sequencing) have been developed that greatly facilitate the identification of miR-mRNA targeting relationships, and these have been applied to the study of liver regeneration [15]. Depending on the miR/mRNA pair, miRs can either cause degradation of the mRNA or lead to the inhibition of its translation [11].

As the name implies, long non-coding RNAs (lncRNAs) are more sizable than miRs, operationally defined as being at least 200 nucleotides in length [16]. Some estimates for the number of lncRNAs encoded in humans exceed 100,000, although the number that are actually transcribed or functional is unknown [16]. LncRNAs resemble protein-coding mRNAs in both their length and nuclear processing (many possess a 5′ cap and 3′ polyadenylation and have introns); however, they are processed less efficiently [16]. LncRNAs are more likely than mRNAs to be transcribed by phosphorylation-dysregulated Pol II (Figure 1B). This can lead to lncRNA transcripts becoming tethered to chromatin, where they are either degraded or accumulate over time [16]. The splicing signals of lncRNAs are also weaker, and therefore lncRNAs are frequently retained in the nucleus due to inefficient splicing [16].

Importantly, nuclear localization does not mean that a given lncRNA is biologically inert. LncRNAs impact gene regulation through a wide array of physical interactions (Figure 2B). LncRNA-chromatin interactions are capable of recruiting or decoying chromatin modifiers, and of interacting directly with the DNA to form DNA-DNA-RNA triplexes and R-loops, both of which have unique effects on gene regulation [16]. LncRNAs are also well known to silence gene expression. This can serve to balance gene dosage, most famously in the silencing of the second mammalian female X chromosome through lncRNA *XIST* in a process termed “X-inactivation” [16,17]. LncRNAs can also act on gene expression through alterations to gene accessibility or recruitment of transcription factors [16]. Finally, lncRNAs have been shown to alter pre-mRNA splicing through direct association with mRNAs or recruitment or decoying of splicing factors [16,18].

Circular RNAs (circRNAs), self-joined RNA molecules, have proven difficult to isolate and characterize due to their lack of the canonical features of mature RNA, such as a polyadenylated 3′ end [19]. However, with continuing advances in sequencing technology, the role of these unique molecules in disease pathology and homeostasis is beginning to be appreciated. There are multiple ways circRNAs can arise in a cell (Figure 1C). Inverted repeat elements or RNA-binding proteins that flank one or more exons can lead to back-splicing. This results in a circular RNA molecule containing either solely exons or both exons and introns [19]. Back-splicing can also occur in lariats created during exon-skipping, leading to a circRNA containing the skipped exon(s) [19]. Lastly, lariats that escape debranching in canonical linear splicing can self-join to become circRNAs containing only introns [19]. The majority of exonic circRNAs are exported to the cytoplasm, while circRNAs containing introns tend to remain in the nucleus [19]. Since these molecules do not have exposed ends like most RNA molecules, they are extremely stable and tend to accumulate with age [19].

Although most circRNAs remain uncharacterized, those that have been studied in-depth have revealed multiple potential mechanisms of action (Figure 2C). The majority of those analyzed thus far have been proposed to function as sponges for miRs, reducing their effective concentration [19]. CircRNAs are also able to decoy RNA-binding proteins, and a few have been shown to bind enzymes and their substrates, promoting colocalization and activity [19]. Although not yet experimentally confirmed, circRNAs may also functionally enhance or recruit proteins [19]. Some circRNAs contain internal ribosome entry sites and AUG codons, making it possible to be translated, which could give rise to unique peptides that would not be possible through canonical RNA processing [19].

In addition to biological functions in their cell of origin, ncRNAs are sometimes secreted from the cell, typically in exosomes. The protection of the exosomal membrane allows ncRNAs to remain relatively stable in the extracellular space, and exosomal ncRNAs have been detected in both the blood and urine of human patients [20]. Given this stability, and the ability of cells to uptake exosomes produced by distal tissues, it has been proposed that exosomal ncRNAs are able to exert biological effects far from their cell of origin [21,22]. The ability to monitor ncRNA levels through the non-invasive collection of body fluids has made exosomal ncRNAs prime candidates to act as biomarkers for various disease states, a topic which will be discussed later in this review.

Numerous studies have found changes in non-coding RNAs in various liver pathologies (see Table 1), a topic this review will seek to examine through current literature on the subject. However, as ncRNAs in humans may number in the hundreds of thousands, the scope of this review is necessarily limited. Emphasis has been placed on RNA species that are highly expressed in the liver, whether at baseline or in proliferative states, or that have experimentally proven impacts on liver cell biology reflected in multiple sources. Examined pathologies and injury states will be mainly those affecting hepatocyte function.

## 3. MiR-122

MiR-122 is by far the most highly expressed microRNA in the liver, and by some estimates constitutes up to 70% of hepatic miRs [49,50]. As such, it comes as no surprise that miR-122 expression is disrupted in diverse liver pathologies including hepatitis C viral infection [23,24] and non-alcoholic steatohepatitis [25,26,27,28]. MiR-122 is transcribed from a noncoding RNA exon on human chromosome 18. mRNAs regulated by miR-122 change in abundance in a circadian fashion, although it is not known how this occurs. The total abundance of miR-122 does not change much from day to night due to its long half-life, despite the fact that miR-122 transcription follows a circadian pattern [49]. One way to reconcile these findings is by invoking a model in which newly synthesized miR-122 molecules constitute a separate, active pool of miRs. The sequence of miR-122 exists downstream of a liver-specific promotor as well as a target site for the binding of hepatocyte nuclear factor 4α (HNF-4α), a transcription factor critical for liver development and function [49]. HNF-4α has been found to be expressed and to bind chromatin in a rhythmic pattern, which could explain the circadian nature of miR-122 expression [51].

An important function of miR-122 in liver pathology relates to its effects on hepatitis C virus (HCV) infection. HCV replicates predominantly in the liver, and infection can lead to chronic liver problems such as cirrhosis and hepatocellular carcinoma (HCC) [24,52]. MiR-122 has multiple potential target sites in the HCV genome, two of which lie in the virus’ 5′-UTR and appear to regulate translation. Translation of the viral genome is stimulated by the binding of miR-122 to these two sites (HCV is a positive-sense single-stranded RNA virus, so proteins are translated directly from the genome) [24,53,54]. When miR-122 is sequestered, and thus unavailable to interact with the viral genome, HCV replication is decreased [23]. MiR-122 was shown to accelerate the formation of ribosomal 48S initiation complexes by promoting the association between the small ribosomal subunit and the HCV genome, indicating at least one mechanism by which miR-122 stimulates viral translation [24].

MiR-122 also impacts non-alcoholic steatohepatitis (NASH), but the literature is conflicted regarding its role. Cheung and colleagues found that anti-miR targeting of miR-122 in cultured cells causes the increased expression of lipogenic genes, thus impacting de novo lipogenesis and contributing to NASH pathology [25]. In contrast, a study by Long and colleagues found increased miR-122 expression in C57BL/6 mice fed a high-fat diet and human hepatoma cell lines supplemented with free fatty acids, both of which are models for non-alcoholic fatty liver disease (NAFLD). Their inhibition of miR-122 in human hepatoma cell lines HepG2 and Huh-7 reduced lipid deposition and triglyceride secretion, processes that contribute to steatohepatitis [26]. A study by Esau and colleagues supports this finding. This group used antisense oligonucleotides to inhibit miR-122 in the livers of mice with diet-induced obesity and found that inhibition of this miR improved hepatic steatosis [27]. The duration of miR-122 loss might be important here, as transient miR-122 inhibition downregulates lipogenic genes and alleviates NASH in vivo, while mice with a germ line or hepatocyte-specific deletion of miR-122 show increased lipid accumulation and a greater risk of NASH [28]. Further experimentation is necessary to elucidate the reasons behind the varied role of miR-122 in NAFLD and NASH.

Finally, miR-122 also plays a role in alcoholic liver disease (ALD). Thus, Satishchandran and colleagues found levels of this miR reduced in the liver of patients diagnosed with ALD, as well as in mice raised on the Lieber-DeCarli diet [55]. Conversely, when the authors over-expressed miR-122 in hepatocytes of transgenic mice and induced hepatic steatosis and fibrosis with ethanol, they observed reduced serum levels of alanine aminotransferase as well as the improvement of the liver phenotype. Remarkably, the horizontal transfer of miR-122 mediated by exosomes from ethanol treated hepatoma cells to monocytes was observed in vitro, and alcohol-fed mice exhibited elevated levels of miR-122 in Kupffer cells and mononuclear cells in vivo. Monocytes thus pre-conditioned in culture were more sensitive to lipopolysaccharide (LPS) treatment and increased production of pro-inflammatory cytokines [56]. These findings suggest, although they do not prove, that the in vivo exosomal transfer of miR-122 could impact the pathogenesis of ALD. 

## 4. MiR-21

Although not as highly expressed in the liver as miR-122, miR-21 impacts liver biology and disease in multiple ways. MiR-21 affects metabolic and regenerative processes that influence pathologies such as NASH and hepatocellular carcinoma (HCC) [29,30,31,32,33]. MiR-21 is located on the forward strand of human chromosome 17. The 3p form is 21 nucleotides long and localizes to the extracellular space, while the 5p form is 22 nucleotides long and can be found in both the cytoplasm and extracellular space [57]. The 5p form is more highly expressed and directly implicated in several pathologies.

MiR-21 is upregulated in human NASH liver samples. Loyer and colleagues proposed a mechanism by which this miR may contribute to disease progression. They found that pharmacological suppression or genetic ablation of miR-21 led to a reduction in NASH features, such as liver cell injury, inflammation, and fibrosis, in mice fed a high-fat or methionine-choline-deficient diet [30,58]. Likewise, when Calo and colleagues employed both whole body as well as hepatocyte-specific ablation of miR-21, they found no metabolic changes in either model in mice fed a standard, low-fat rodent diet, but observed that miR-21 deficient mice displayed improved glucose tolerance and steatosis on a high fat diet [59]. Based on previous work that found a negative correlation between expression of PPARα (a key regulator of fatty acid oxidation) and NASH severity, miR-21 was also suppressed in PPARα null mice by Loyer and colleagues [29,30,60]. Mir-21 suppression was found to have less of a protective effect against NASH in these mice than in controls, suggesting that PPARα is part of miR-21’s mechanism of action [30]. Rodrigues and colleagues recapitulated many of these findings, showing that miR-21 null mice display fewer features of NASH. This group also showed that wild-type mice fed a “fast food diet” to induce features of metabolic pathologies had higher miR-21 and reduced PPARα expression in the liver [61]. However, miR-21 does not only function in fatty liver disease. The deletion of miR-21 from hepatocytes lead to improved outcomes regarding insulin sensitivity and lipid metabolism in mice challenged with a high-fat diet, potentially tying miR-21 to metabolic disorders in general [59]. Interestingly, the relationship between miR-21 and the PPARs appears to be complex. Thus, in addition to the aforementioned regulation of PPARα by miR-21, a recent report suggests that PPARγ directly suppresses the transcription of the miR-21 gene [62].

In addition to its effects on metabolism, miR-21 can module hepatocyte proliferation and liver regeneration. MiR-21 expression is rapidly upregulated following two-thirds partial hepatectomy (PH) in mice, which appears to accelerate cyclin D1 translation to allow hepatocytes to move through the cell cycle more rapidly [32]. However, miR-21 is also more highly expressed in ethanol-fed rat livers despite a reduction in liver regenerative ability. The inhibition of miR-21 using an anti-miR restores regenerative ability in these rats [31]. The genetic deletion of miR-21 in mice has also been shown to promote HCC oncogenesis. The deletion of miR-21 dysregulates several oncogenes and immune factors, which could create a pro-tumor environment in the liver despite the loss of miR-21’s impact on cell proliferation. MiR-21’s effect on HCC also appears to be dependent on the presence of other cancer-associated mutations [33]. These seemingly contradictory findings suggest that miR-21’s effect on hepatocyte proliferation and liver regeneration is context-specific.

## 5. The Let-7 Family

As one of the first microRNAs to be discovered, the “founding member” of the let-7 family was originally given the name “lethal-7”, a nomenclature which has remained despite its deviation from modern miR naming conventions [63]. The precursor sequence is 88 nucleotides long and is located on the reverse strand of human chromosome 3. Vertebrates express multiple let-7 family members, with the most prevalent in the human liver being let-7a-5p, let-7f-5p, let-7g-5p, and let-7b-5p [50]. However, even less prevalent members can impact liver phenotypes.

Expression levels of several let-7 family members are altered in response to liver injury. In mice given an overdose of acetaminophen, hepatic let-7b and let-7c were downregulated, let-7g was upregulated in the plasma, and let-7d was upregulated in both the liver and plasma [37]. In rats, let-7f and let-7b were downregulated in 45% small volume liver grafts. Multiple let-7 members showed increased expression in the liver one day after a 50% partial hepatectomy and then decreased expression by day three following the surgery. Genes encoding for multiple cyclins and cyclin-dependent kinases were upregulated following partial hepatectomy, and several members of the let-7 family have been proposed to target these genes, revealing a possible mechanism by which this family of miRs might modulate liver regeneration [36]. However, this mechanism needs to be experimentally validated. In addition, experimental cholestasis induced by bile duct ligation in mice leads to reduced expression of let-7a [64]. Likewise, the hormone secretin, produced by duodenal enteroendocrine S cells, stimulates the proliferation of biliary epithelial cells, and in conditions of bile duct ligation, is also produced by large cholangiocytes [65]. Interestingly, secretin appears to exert part of its effect via the inhibition of let-7a, as the inhibition of let-7a caused the increased proliferation of large cholangiocytes in vitro [65].

While the full extent of let-7 family function has yet to be elucidated in the healthy liver, the highly proliferative nature of cancer cells offers some clues as to how these miRs function in hepatocyte proliferation. Let-7a-5p, let-7b-5p, and let-7g-5p have all been shown to inhibit the progression of hepatic tumors, although they seem to do so through different mechanisms. A 2020 study by Liu and colleagues showed that let-7a-5p reduces proliferation and invasion in human HCC cell lines and sensitizes cells to the anti-tumor effects of the multi-kinase inhibitor Sorafenib. This was proposed to occur through the downregulation of basic leucine zipper and W2 domains 2 (BZW2) [34], a protein-coding gene predicted to function in cell differentiation [66]. Let-7a-5p targets the 3′ UTR of BZW2 [34]. Pan and colleagues explored the role of let-7a in cholangiocarcinoma, the less common but highly deadly liver cancer. They found that let-7a (along with miR-1182) negatively regulated the pro-tumor gene NUAK1. Let-7a and miR-1182 downregulate NUAK1 mRNA, but have reduced expression in cholangiocarcinoma, leading to aberrant NUAK1 expression. The addition of let-7a and/or miR-1182 mimics to human cholangiocarcinoma cell lines resulted in increased autophagy [35]. Another family member, let-7b, also functions as a tumor suppressor. Hui and colleagues found that the addition of a let-7b mimic reduced the proliferation of human HCC cell lines. This occurs through the upregulation of CDKN1A, the gene encoding the tumor suppressor p21, which has let-7b binding sites at the 3′ UTR. The in vitro depletion of p21 ameliorated the effect of let-7b mimic addition [38]. 

Let-7g has also been shown to suppress HCC, although the proposed mechanisms by which it acts as a tumor suppressor are more varied than those of other family members. A 2014 study by Chen and colleagues found that transfecting human HCC cell lines with a vector containing the let-7g precursor sequence impeded the cells’ invasive ability. Cells transfected with the let-7g vector were also more likely to apoptose or enter cell cycle arrest during G1 than control cells. Transfected cells displayed downregulation of MAPK pathway members and alterations in factors contributing to the epithelial-mesenchymal transition, such as Snail, N-cadherin, E-cadherin, and HMGA2A, suggesting that let-7g may target genes associated with these pathways [40]. Ji and colleagues similarly found that the overexpression let-7g in the human HCC cell line huH-1 reduced cell migration and proliferation. This group found that let-7g overexpression decreased levels of soluble collagens, and that COL1A2 was a direct target of let-7g, proposing another mechanism by which the miR may alter cancer cell behavior [41]. Yet another mechanism of let-7g HCC regulation was explored by Lan and colleagues. Their study suggested that let-7g has a more direct effect on tumorigenesis by downregulating the expression of the proto-oncogene c-Myc and the upregulating of the tumor suppressor p16^INK4A^*,* as evidenced through overexpression of the miR. Accordingly, let-7g inhibition had the opposite effect [42].

Cancer is not the only liver pathology modulated by let-7g. Chou and colleagues found that hepatitis C virus (HCV) patients with a sustained virologic response (meaning that the virus remained undetectable 12 weeks or more after the cessation of treatment) had higher let-7g expression in serum and the liver [43]. Additionally, transfection of a let-7g mimic into Ava.5 cells (a HuH7-based cell line containing sub-genomic HCV replicons) notably reduced the HCV viral load. Let-7g expression was increased by treatment with interferon α-2a or ribavirin, with an additive effect when both therapies were used [43]. The increased expression was modulated by the binding of activator protein-1 (AP-1) to the let-7g promotor and occurred through p38 pathway signaling. Additionally, HCV infection increased levels of Lin28A and Lin28B, which both inhibit let-7g expression. This could indicate a mechanism by which HCV suppresses let-7g expression to allow for viral replication [43]. Let-7b has a similar suppressive effect on HCV replication. Cheng and colleagues showed that in a cell culture model of HCV, transfection with let-7b reduced the infectivity of HCV. Let-7b was found to be able to interact with the HCV genome directly by participating in a miRISC complex containing Ago2 and HCV RNA. Furthermore, interferon α-2a was found to suppress viral replication in tandem with let-7b [39]. In summary, multiple let-7 family members act as tumor suppressors and let-7b and let-7g reduce HCV replication. Let-7 family members are also altered during liver injury, but any direct effects on injury and regeneration have yet to be experimentally validated.

## 6. MiR-451a and MiR-144

MiR-451a is encoded by a 22-nucleotide sequence from the reverse strand of human chromosome 17. MiR-144 is created from an 86-nucleotide sequence fewer than 100 nucleotides downstream of the miR-451a sequence [57,67]. Both miRs are typically secreted into the extracellular space [57]. In addition to being closely spatially linked, miR-451a and miR-144 are also frequently expressed in similar ways in liver pathology, hence their inclusion together in this section. 

In a recent study by Loukachov and colleagues, miR-451a, miR-144-3p, and miR-144-5p were three of only five miRs that displayed altered expression in both the plasma and liver of chronic hepatitis B patients, the others being miR-182-5p and miR-206 [44]. In addition, both miR-144 and miR-451a have been found to suppress the progression of HCC, possibly in conjunction. Both miRs are typically decreased in HCC patients. The presence of miR-451a or miR-144 is correlated with a better prognosis, and overexpression of either can increase the survival time of mice with HCC [45]. Work by Zhao and colleagues showed that this may occur due to miR modulation of the tumor microenvironment. Overexpression of miR-451a and miR-144 reduced angiogenesis, increased CD8+ T cell infiltration, reduced tumor-associated Tregs, and increased the expression of anti-tumor (or M1) markers on tumor-associated macrophages. This effect appears to occur in a paracrine fashion through the regulation of cytokine production, including hepatocyte growth factor and macrophage migration inhibitory factor, predicted targets of miR-144 and miR-451a [45]. Lv and colleagues found that the long non-coding RNA ZSCAN16 antisense RNA 1 (ZSCAN16-AS1) can bind and decoy miR-451a, reducing its ability to act as a tumor suppressing miR [46].

## 7. MALAT1

Another important player in liver pathologies is MALAT1. Although originally named for its role in lung cancer (metastasis-associated lung adenocarcinoma transcript 1), this lncRNA can impact liver diseases as well. MALAT1 is transcribed from an 8779-nucleotide gene on chromosome 11. There are three transcript variants containing between one and three exons [66]. For the mature MALAT1 molecule to be formed, a small ncRNA is cleaved from the 3′ end of the transcript product. This processing step leaves MALAT1 without the poly(A) tail that many lncRNAs possess. However, it has a 3′ triple helical structure which stabilizes the transcript [66]. MALAT1 is retained in the nucleus, where it associates primarily with transcriptionally active genes. It is implicated in alternative splicing, alteration of protein functionality, and the decoying of other ncRNAs [47,66]. Several proteins regulate MALAT1; for instance, the serine/arginine splicing factor 1 (SRSF1) downregulates MALAT1 expression [47]. Conversely, yes-associated protein (YAP) promotes the expression of MALAT1, in part by decreasing the nuclear retention of SRSF1 [47]. Specific proteins 1 and 3 (SP1/3) can also upregulate MALAT1 expression by binding upstream of its gene [47].

MALAT1 is overexpressed in HCC, where it drives proliferation and migration along with reduced apoptosis in cancerous cells [47]. Multiple oncogenic mechanisms have been proposed for MALAT1. It acts as a sponge for a number of miRs, including miR-22 (a regulator of the epithelial-mesenchymal transition transcription factor SNAI1) [47]. MALAT1 also activates the mTORC1-4EBP1 axis, which promotes glycolysis, a hallmark of tumor cell metabolism [47]. Increased hepatocyte growth factor promotes the expression of MALAT1, indicating that this lncRNA may also have a role in liver regeneration [47]. MALAT1 is linked to lipid accumulation in the liver, a process that often leads to NAFLD or NASH. One proposed mechanism involves SREBP-1c (sterol regulator element binding protein 1c), a protein that activates the PNPLA3 gene to increase lipid accumulation [68], which is stabilized by MALAT1 [48]. MALAT1 has also been implicated in fibrosis and liver regeneration, which a review by Lu and colleagues covers in more detail [47].

## 8. Other Notable ncRNAs

With the enormous number of ncRNAs expressed in the liver, high-throughput screens to identify species of interest are imperative to increasing our understanding of their functions in liver injury and regeneration. One such screen was developed by Zahm and colleagues and used over 30,000 tough decoy miR inhibitors in a mouse model of toxic liver injury to identify miRs that modulate hepatic repopulation. Thousands of tough decoys showed altered abundance in the recovered liver, and pairwise interactions were mapped across 171 miR distinct binding sites. While far too many tough decoys showed altered abundance to list their targets individually here, of particular interest were miR-214-3p and miR-374b-5p, which were targeted by the most depleted and most enriched tough decoys, respectively [69].

While many pathways and networks including ncRNAs are altered in HCC, of special note are some of the connections discovered by Chen and colleagues. This group used a bioinformatics approach to identify “hub genes” among differentially expressed genes in HCC, and then identify ncRNAs that are predicted to target those genes or act on other ncRNAs that target them. From 199 miRs, 106 lncRNA- miRNA pairs, and 43,701 circRNAs, nineteen ncRNAs and five mRNAs were chosen as important network members in HCC. Of these, there were eight human miRs (miR-26a-5p, miR-10a-5p, miR-150-5p, miR-486-5p, miR-19b-3p, miR-23b-3p, miR-214-5p, and miR-651-5p), five lncRNAs (DLGAP1-AS1, TYMSOS, GAS5, LINC00665, and ZFAS1), and six human circRNAs (circ_0082333, circ_0003209, circ_0034049, circ_0020396, circ_008128, and circ_0030051) [70]. The mRNAs targeted by this network, MCM4, MCM6, MCM7, CDC6, and GINS1, all function in replication initiation as per the NIH Gene database [66,70]. Of the pathways involved in this network, the authors identified the ZFAS1/has-miR-150-5p/GINS1 axis as being especially novel. By their model, GINS1 acts as an oncogene, promoting progression through the cell cycle, but is regulated by miR-150-5p in humans. However, miR-150-5p can be sponged by lncRNA ZFAS1, negating its anti-tumor effects [70]. 

In addition to this network, several other ncRNAs are implicated in liver tumor cell replication. NIHCOLE, an lncRNA identified by Unfried and colleagues, is associated with proliferation and reduced apoptosis in HCC cells. Depleting NIHCOLE led to a decrease in non-homologous end-joining and accumulation in DNA damage in human HCC cells. Therefore, it is proposed that NIHCOLE functions to prevent DNA damage from reaching an unsustainable level in cancerous cells [71]. LINC00205, another lncRNA, has also been shown to contribute to oncogenic proliferation. Cheng and colleagues found that this lncRNA can act as a sponge for miR-26a-5p, which led to increased CDK6 expression and reduced cell cycle arrest rates for cells highly expressing LINC00205 [72]. A recent review by Meng and colleagues identified 23 circRNAs that are upregulated in HCC, and 12 circRNAs that are downregulated and could function as tumor suppressors [73]. Of particular note in the oncogenic circRNAs are circβ-catenin, a circRNA transcribed from β-catenin, a known oncogene, and circHIPK3, the fourth most highly expressed circRNA in the liver [73,74]. Notable among the tumor suppressive circRNAs are circZKSCAN1 and circC3P1, which are the second and thirteenth most expressed circRNAs in the liver, respectively [73,74]. 

While many ncRNAs have been implicated in tumorigenesis within the liver, many others function in liver injury and regeneration in other pathological contexts. As mentioned previously in the let-7 family section, Chen and colleagues profiled miRs in rat livers in small volume grafts and the remaining liver sections. In addition to changes in let-7 family members, they found miR-26a to be downregulated in the 45% volume grafts, and miRs 22a, 26a, and 30b were downregulated in the remaining liver tissue. Similarly to let-7 members, these miRs increased in expression the day following the procedure and then decreased substantially, indicating that they may be regulated dynamically at different stages of injury and healing. All miRs listed above are predicted by bioinformatics to negatively regulate cell cycle genes such as cyclins and cyclin-dependent kinases. Cyclin E2 was experimentally determined to be inhibited by miR-26a [36]. The lncRNA MAYA has been shown to promote hepatocyte senescence, a process linked to the pathogenesis of NAFLD, in HFD-fed mice through the inhibition of YAP (yes-associated protein) [75]. YAP is a protein in the Hippo signaling pathway and has been implicated in the regulation of lipid accumulation and iron overloading in hepatocytes, the latter of which has been linked to cellular senescence [75]. The long non-coding RNA ARSR has been shown to promote cholesterol biosynthesis, leading to increases in NAFLD/NASH severity in mice fed a high cholesterol diet [76]. Similarly, lnc-HC has been shown to inhibit the metabolism of HFD- and high cholesterol diet-fed mice [76]. H19 also functions in NAFLD pathogenesis, but this lncRNA functions by promoting transcription and mRNA stability for genes contributing to steatosis [76]. Finally, Blnc1 promotes NAFLD by increasing triglyceride synthesis through scaffolding of the gene EDF1 [76].

## 9. Circulating ncRNAs as Biomarkers

While many ncRNAs exert regulatory effects directly in their cells of origin, others are secreted into the extracellular space or released in exosomes. The expression levels of dozens of ncRNAs have been shown to be altered in plasma or other distal sites in response to liver disease or injury. Therefore, ncRNAs have been proposed as potential biomarkers to detect liver disease in its earliest stages. Table 2 lists ncRNAs that may function as disease prognosticators.

## 10. Conclusions

Once undetectable or considered unimportant, the recent focus on ncRNAs has revealed their multifaced effects on human health. However, there is still a long way to go until the knowledge gleaned from these studies can be brought to clinical practice for the treatment of chronic liver disease or HCC. Current clinical trials involving ncRNAs focus on their diagnostic potential rather than employing them as therapies, and even then, it is rare to see a clinical trial for a species of ncRNA other than miRs. Additionally, some of the lesser-studied species of ncRNA, such as PIWI-interacting RNAs (piRNAs), small nucleolar RNAs (snoRNAs) and tRNA-derived small RNAs (tRFs) have only recently attracted widespread attention [82]. As our view of ncRNAs continues to expand, perhaps one day they will take their place in the therapeutic arsenal of gastroenterologists worldwide.

## 11. Methodology

In July 2022, PubMed was searched using the input “(liver) AND ((injury) OR (damage) OR (regeneration))”. Filters were set to look for full text sources published within the last year. A list of 9310 results was downloaded and imported into SWIFT Review (desktop version 1.43, build 10535: 02 Feb 2022). Based on relevance to human liver pathology, 62 training documents were chosen from the imported list (42 included and 20 excluded) to create a sorted list of most relevant studies. Word clouds were generated for all results and for a list of top results by generated relevancy scores, and topics for this review were chosen based on these results.

To establish potentially relevant miRs, a list of 2657 microRNAs from the human liver as determined by next generation sequencing was downloaded from TissueAtlas2 [50]. MicroRNAs with an expression mean of 0 were removed, leaving 2008 potential miRs of interest. A relative expression percentage for each miR was generated by calculating the expression mean with the summed expression means of all listed miRs. MicroRNAs with a relative expression percentage of 1% or greater were searched on PubMed. Additional miRs of interest were determined from the results of these searches. Relevant lncRNAs and circRNAs were identified through relationships to miRs and through PubMed searches.

## Figures and Tables

**Table 1:** Summary of ncRNA-associated pathologies and functions detailed in the main text.

ncRNA	Disease Association(s)	Other Notable Function(s)	Citations
miR-122	Hepatitis C		[23,24,25,26,27,28]
	NASH		
miR-21	NASH	Limits regenerative ability	[29,30,31,32,33]
	Hepatocellular carcinoma		
let-7a	Hepatocellular carcinoma	Altered following partial hepatectomy	[34,35,36]
	Cholangiocarcinoma		
let-7b	Hepatitis C	Downregulated in small volume liver grafts	[36,37,38,39]
	Acetaminophen overdose	Altered following partial hepatectomy	
	Hepatocellular carcinoma		
let-7c	Acetaminophen overdose	Altered following partial hepatectomy	[36,37]
let-7d	Acetaminophen overdose	Altered following partial hepatectomy	[36,37]
let-7e		Altered following partial hepatectomy	[36]
let-7f		Downregulated in small volume liver grafts	[36]
		Altered following partial hepatectomy	
let-7g	Hepatitis C	Altered following partial hepatectomy	[36,40,41,42,43]
	Hepatocellular carcinoma		
let-7i		Altered following partial hepatectomy	[36]
miR-451a	Chronic hepatitis B		[44,45]
	Hepatocellular carcinoma		
miR-144	Chronic hepatitis B		[44,45]
	Hepatocellular carcinoma		
ZSCAN16-AS1 (lncRNA)	Hepatocellular carcinoma		[46]
MALAT1 (lncRNA)	Hepatocellular carcinoma NAFLD/NASH Fibrosis	Liver regeneration	[47,48]

**Table 2:** Potential ncRNA biomarkers from blood, plasma, or serum.

ncRNA	Disease Association	Citations
miR-1224	Acetaminophen injury *	[37]
miR-124		
miR-125a-3p		
miR-125b-5p		
miR-133a		
miR-133b		
miR-135a		
miR-202-3p		
miR-205		
miR-23a		
miR-26a		
miR-486		
miR-710		
miR-711		
miR-712		
miR-720		
miR-721		
miR-193	Acetaminophen injury ^, Cirrhosis *	[37,77]
let-7d	Acetaminophen injury ^	[37]
let-7g		
miR-101b		
miR-107		
miR-130a		
miR-148a		
miR-15a		
miR-19b		
miR-22		
miR-27b		
miR-294		
miR-29a		
miR-29c		
miR-30a		
miR-30c		
miR-30e		
miR-365		
miR-574-5p		
miR-680		
miR-685		
miR-122	Acetaminophen injury ^, Chronic hepatitis B ^	[37,44]
miR-192	Acetaminophen injury ^, NAFLD ^, NASH ^	[37,78]
miR-21		
miR-29b		
let-7e-5p	Chronic hepatitis B *	[44]
miR-125a-5p		
miR-144-3p		
miR-144-5p		
miR-182-5p		
miR-190a-5p		
miR-454-3p		
miR-7-5p		
miR-99b-5p		
miR-183-5p	Chronic hepatitis B *, NAFLD ^, NASH ^	[44,78]
miR-215-5p	Chronic hepatitis B ^	[44]
miR-224-5p		
miR-320a		
miR-335-5p		
miR-584-5p		
miR-625-3p		
miR-146a-5p		
miR-206		
miR-4433bb-3p		
MEG3 (lncRNA)	Chronic hepatitis B-associated fibrosis *	[79]
miR-152	Cirrhosis *	[77]
miR-574-3p		
miR-483-5p	Cirrhosis *, Acetaminophen injury *, Chronic hepatitis B ^	[37,44,77]
miR-451a	Cirrhosis ^, Acetaminophen injury *, Chronic hepatitis B *	
miR-222-3p	Cirrhosis ^	[77]
SCARNA10	Fibrosis ^, cirrhosis ^	[80]
circ_000520	HCC *	[81]
circ_001565		
cSMARCA5 (circRNA)		
miR-101-3p		[77]
circ_0003731	HCC ^	[81]
circ_0008043		
circ_00244		
circ_0064428		
circ_0088030		
circ_104075		
circ-ZEB1.33		
let-7a-5p		[77]
miR-128		
miR-139-5p		
miR-320d		
miR-382-5p		
miR-423-5p	HCC ^, Chronic hepatitis B ^	[44,77]
miR-126-5p	NAFLD ^, NASH ^	[78]
miR-151a-3p		
miR-15b-3p		
miR-4449		
miR-214-3p	Steatosis *	[77]
miR-95		
miR-424-5p	Viral infection *, HCC *	[77]
miR-410	Viral infection ^	[77]

* ncRNA is reduced in circulation; ^ ncRNA is increased in circulation.

## Glossary of Abbreviations

Ago	Argonaute
AP-1	activator protein-1
circRNA	circular RNA
CLIP-Seq	cross-linking immunoprecipitation followed by high throughput sequencing
HCC	hepatocellular carcinoma
HBV	hepatitis B virus
HCV	hepatitis C virus
HFD	high fat diet
HNF-4α	hepatocyte nuclear factor 4α
lncRNA	long non-coding RNA
MALAT1	metastasis-associated lung adenocarcinoma transcript 1
MIF	migration inhibitory factor
miR	microRNA
miRISC	microRNA-induced silencing complex
MRE	microRNA recognition element
NAFLD	non-alcoholic fatty liver disease
NASH	non-alcoholic steatohepatitis
ncRNA	non-coding RNA
PACT	protein kinase RNA activator
piRNA	PIWI-interacting RNA
pre-miR	precursor microRNA
pri-miR	primary microRNA
RBP	RNA-binding protein
SF	splicing factor
snoRNA	small nucleolar RNA
SRSF1	serine/arginine splicing factor 1
TRBP	transactivation response element RNA-binding protein
tRF	tRNA-derived small RNA
YAP	yes-associated protein
ZSCAN16-AS1	ZSCAN16 antisense RNA 1 (long non-coding RNA)

## References

[1] Cheemerla S., Balakrishnan M. (2021). Global Epidemiology of Chronic Liver Disease. Clin. Liver Dis..

[2] Cirrhosis. https://www.nhs.uk/conditions/cirrhosis/#:~:text=Cirrhosis%20is%20scarring%20(fibrosis)%20of,the%20liver%2C%20such%20as%20hepatitis.

[3] Mengshol J.A., Golden-Mason L., Rosen H.R. (2007). Mechanisms of Disease: HCV-induced liver injury. Nat. Clin. Pract. Gastroenterol. Hepatol..

[4] Suhail M., Abdel-Hafiz H., Ali A., Fatima K., Damanhouri G.A., Azhar E., Chaudhary A.G., Qadri I. (2014). Potential mechanisms of hepatitis B virus induced liver injury. World J. Gastroenterol..

[5] Byrne C.D., Targher G. (2015). NAFLD: A multisystem disease. J. Hepatol..

[6] Diseases, NIDDK. Definition & Facts of NAFLD & NASH. https://www.niddk.nih.gov/health-information/liver-disease/nafld-nash/definition-facts.

[7] McGlynn K.A., Petrick J.L., London W.T. (2015). Global epidemiology of hepatocellular carcinoma: An emphasis on demographic and regional variability. Clin. Liver Dis..

[8] Holczbauer A., Wangensteen K.J., Shin S. (2022). Cellular origins of regenerating liver and hepatocellular carcinoma. JHEP Rep..

[9] Michalopoulos G.K., Bhushan B. (2021). Liver regeneration: Biological and pathological mechanisms and implications. Nat. Rev. Gastroenterol. Hepatol..

[10] Friedman J.R., Kaestner K.H. (2011). On the origin of the liver. J. Clin. Investig..

[11] O’Brien J., Hayder H., Zayed Y., Peng C. (2018). Overview of MicroRNA Biogenesis, Mechanisms of Actions, and Circulation. Front. Endocrinol..

[12] Conrad T., Ntini E., Lang B., Cozzuto L., Andersen J.B., Marquardt J.U., Ponomarenko J., Tartaglia G.G., Vang Orom U.A. (2020). Determination of primary microRNA processing in clinical samples by targeted pri-miR-sequencing. RNA.

[13] Shukla G.C., Singh J., Barik S. (2011). MicroRNAs: Processing, Maturation, Target Recognition and Regulatory Functions. Mol. Cell. Pharmacol..

[14] Choo K.B., Soon Y.L., Nguyen P.N., Hiew M.S., Huang C.J. (2014). MicroRNA-5p and -3p co-expression and cross-targeting in colon cancer cells. J. Biomed. Sci..

[15] Schug J., McKenna L.B., Walton G., Hand N., Mukherjee S., Essuman K., Shi Z., Gao Y., Markley K., Nakagawa M. (2013). Dynamic recruitment of microRNAs to their mRNA targets in the regenerating liver. BMC Genom..

[16] Statello L., Guo C.J., Chen L.L., Huarte M. (2021). Gene regulation by long non-coding RNAs and its biological functions. Nat. Rev. Mol. Cell Biol..

[17] Sahakyan A., Yang Y., Plath K. (2018). The Role of Xist in X-Chromosome Dosage Compensation. Trends Cell Biol..

[18] Pisignano G., Ladomery M. (2021). Epigenetic Regulation of Alternative Splicing: How LncRNAs Tailor the Message. Noncoding RNA.

[19] Kristensen L.S., Andersen M.S., Stagsted L.V.W., Ebbesen K.K., Hansen T.B., Kjems J. (2019). The biogenesis, biology and characterization of circular RNAs. Nat. Rev. Genet..

[20] Xu Y.X., Pu S.D., Li X., Yu Z.W., Zhang Y.T., Tong X.W., Shan Y.Y., Gao X.Y. (2022). Exosomal ncRNAs: Novel therapeutic target and biomarker for diabetic complications. Pharmacol. Res..

[21] Isaac R., Reis F.C.G., Ying W., Olefsky J.M. (2021). Exosomes as mediators of intercellular crosstalk in metabolism. Cell Metab..

[22] Wang D., Zhang W., Zhang C., Wang L., Chen H., Xu J. (2022). Exosomal non-coding RNAs have a significant effect on tumor metastasis. Mol. Ther. Nucleic Acids.

[23] Jopling C.L., Yi M., Lancaster A.M., Lemon S.M., Sarnow P. (2005). Modulation of hepatitis C virus RNA abundance by a liver-specific MicroRNA. Science.

[24] Henke J.I., Goergen D., Zheng J., Song Y., Schuttler C.G., Fehr C., Junemann C., Niepmann M. (2008). microRNA-122 stimulates translation of hepatitis C virus RNA. EMBO J..

[25] Cheung O., Puri P., Eicken C., Contos M.J., Mirshahi F., Maher J.W., Kellum J.M., Min H., Luketic V.A., Sanyal A.J. (2008). Nonalcoholic steatohepatitis is associated with altered hepatic MicroRNA expression. Hepatology.

[26] Long J.K., Dai W., Zheng Y.W., Zhao S.P. (2019). miR-122 promotes hepatic lipogenesis via inhibiting the LKB1/AMPK pathway by targeting Sirt1 in non-alcoholic fatty liver disease. Mol. Med..

[27] Esau C., Davis S., Murray S.F., Yu X.X., Pandey S.K., Pear M., Watts L., Booten S.L., Graham M., McKay R. (2006). miR-122 regulation of lipid metabolism revealed by in vivo antisense targeting. Cell Metab..

[28] Huang R., Duan X., Fan J., Li G., Wang B. (2019). Role of Noncoding RNA in Development of Nonalcoholic Fatty Liver Disease. Biomed. Res. Int..

[29] van Raalte D.H., Li M., Pritchard P.H., Wasan K.M. (2004). Peroxisome proliferator-activated receptor (PPAR)-alpha: A pharmacological target with a promising future. Pharm. Res..

[30] Loyer X., Paradis V., Henique C., Vion A.C., Colnot N., Guerin C.L., Devue C., On S., Scetbun J., Romain M. (2016). Liver microRNA-21 is overexpressed in non-alcoholic steatohepatitis and contributes to the disease in experimental models by inhibiting PPARalpha expression. Gut.

[31] Juskeviciute E., Dippold R.P., Antony A.N., Swarup A., Vadigepalli R., Hoek J.B. (2016). Inhibition of miR-21 rescues liver regeneration after partial hepatectomy in ethanol-fed rats. Am. J. Physiol. Gastrointest. Liver Physiol..

[32] Ng R., Song G., Roll G.R., Frandsen N.M., Willenbring H. (2012). A microRNA-21 surge facilitates rapid cyclin D1 translation and cell cycle progression in mouse liver regeneration. J. Clin. Investig..

[33] Correia de Sousa M., Calo N., Sobolewski C., Gjorgjieva M., Clement S., Maeder C., Dolicka D., Fournier M., Vinet L., Montet X. (2021). Mir-21 Suppression Promotes Mouse Hepatocarcinogenesis. Cancers.

[34] Liu L., Zhao J., Peng Y., Yang M., Zhang L., Jin X. (2020). miR-let-7a-5p Inhibits Invasion and Migration of Hepatoma Cells by Regulating BZW2 Expression. Onco Targets Ther..

[35] Pan X., Wang G., Wang B. (2021). MicroRNA-1182 and let-7a exert synergistic inhibition on invasion, migration and autophagy of cholangiocarcinoma cells through down-regulation of NUAK1. Cancer Cell Int..

[36] Chen X., Murad M., Cui Y.Y., Yao L.J., Venugopal S.K., Dawson K., Wu J. (2011). miRNA regulation of liver growth after 50% partial hepatectomy and small size grafts in rats. Transplantation.

[37] Wang K., Zhang S., Marzolf B., Troisch P., Brightman A., Hu Z., Hood L.E., Galas D.J. (2009). Circulating microRNAs, potential biomarkers for drug-induced liver injury. Proc. Natl. Acad. Sci. USA.

[38] Hui L., Zheng F., Bo Y., Sen-Lin M., Ai-Jun L., Wei-Ping Z., Yong-Jie Z., Lei Y. (2020). MicroRNA let-7b inhibits cell proliferation via upregulation of p21 in hepatocellular carcinoma. Cell Biosci..

[39] Cheng J.C., Yeh Y.J., Tseng C.P., Hsu S.D., Chang Y.L., Sakamoto N., Huang H.D. (2012). Let-7b is a novel regulator of hepatitis C virus replication. Cell. Mol. Life Sci..

[40] Chen K.J., Hou Y., Wang K., Li J., Xia Y., Yang X.Y., Lv G., Xing X.L., Shen F. (2014). Reexpression of Let-7g microRNA inhibits the proliferation and migration via K-Ras/HMGA2/snail axis in hepatocellular carcinoma. Biomed. Res. Int..

[41] Ji J., Zhao L., Budhu A., Forgues M., Jia H.L., Qin L.X., Ye Q.H., Yu J., Shi X., Tang Z.Y. (2010). Let-7g targets collagen type I alpha2 and inhibits cell migration in hepatocellular carcinoma. J. Hepatol..

[42] Lan F.F., Wang H., Chen Y.C., Chan C.Y., Ng S.S., Li K., Xie D., He M.L., Lin M.C., Kung H.F. (2011). Hsa-let-7g inhibits proliferation of hepatocellular carcinoma cells by downregulation of c-Myc and upregulation of p16(INK4A). Int. J. Cancer.

[43] Chou W.W., Huang C.F., Yeh M.L., Tsai Y.S., Hsieh M.Y., Huang C.I., Huang J.F., Tsai P.C., Hsi E., Juo S.H. (2016). MicroRNA let-7g cooperates with interferon/ribavirin to repress hepatitis C virus replication. J. Mol. Med..

[44] Loukachov V.V., van Dort K.A., Maurer I., Takkenberg R.B., de Niet A., Reesink H.W., Willemse S.B., Kootstra N.A. (2022). Identification of Liver and Plasma microRNAs in Chronic Hepatitis B Virus infection. Front. Cell. Infect. Microbiol..

[45] Zhao J., Li H., Zhao S., Wang E., Zhu J., Feng D., Zhu Y., Dou W., Fan Q., Hu J. (2021). Epigenetic silencing of miR-144/451a cluster contributes to HCC progression via paracrine HGF/MIF-mediated TAM remodeling. Mol. Cancer.

[46] Lv C., Wan Q., Shen C., Wu H., Zhou B., Wang W. (2021). Long noncoding RNA ZSCAN16AS1 promotes the malignant properties of hepatocellular carcinoma by decoying microRNA451a and consequently increasing ATF2 expression. Mol. Med. Rep..

[47] Lu J., Guo J., Liu J., Mao X., Xu K. (2021). Long Non-coding RNA MALAT1: A Key Player in Liver Diseases. Front. Med..

[48] Yan C., Chen J., Chen N. (2016). Long noncoding RNA MALAT1 promotes hepatic steatosis and insulin resistance by increasing nuclear SREBP-1c protein stability. Sci. Rep..

[49] Jopling C. (2012). Liver-specific microRNA-122: Biogenesis and function. RNA Biol..

[50] Keller A., Groger L., Tschernig T., Solomon J., Laham O., Schaum N., Wagner V., Kern F., Schmartz G.P., Li Y. (2022). miRNATissueAtlas2: An update to the human miRNA tissue atlas. Nucleic Acids Res..

[51] Qu M., Duffy T., Hirota T., Kay S.A. (2018). Nuclear receptor HNF4A transrepresses CLOCK:BMAL1 and modulates tissue-specific circadian networks. Proc. Natl. Acad. Sci. USA.

[52] Axley P., Ahmed Z., Ravi S., Singal A.K. (2018). Hepatitis C Virus and Hepatocellular Carcinoma: A Narrative Review. J. Clin. Transl. Hepatol..

[53] Revie D., Salahuddin S.Z. (2011). Human cell types important for hepatitis C virus replication in vivo and in vitro: Old assertions and current evidence. Virol. J..

[54] Payne S. (2017). Introduction to RNA Viruses. Viruses.

[55] Satishchandran A., Ambade A., Rao S., Hsueh Y.C., Iracheta-Vellve A., Tornai D., Lowe P., Gyongyosi B., Li J., Catalano D. (2018). MicroRNA 122, Regulated by GRLH2, Protects Livers of Mice and Patients from Ethanol-Induced Liver Disease. Gastroenterology.

[56] Momen-Heravi F., Bala S., Kodys K., Szabo G. (2015). Exosomes derived from alcohol-treated hepatocytes horizontally transfer liver specific miRNA-122 and sensitize monocytes to LPS. Sci. Rep..

[57] The R.C., Petrov A.I., Kay S.J.E., Kalvari I., Howe K.L., Gray K.A., Bruford E.A., Kersey P.J., Cochrane G., Finn R.D. (2017). RNAcentral: A comprehensive database of non-coding RNA sequences. Nucleic Acids Res..

[58] Kirsch R., Clarkson V., Shephard E.G., Marais D.A., Jaffer M.A., Woodburne V.E., Kirsch R.E., Hall Pde L. (2003). Rodent nutritional model of non-alcoholic steatohepatitis: Species, strain and sex difference studies. J. Gastroenterol. Hepatol..

[59] Calo N., Ramadori P., Sobolewski C., Romero Y., Maeder C., Fournier M., Rantakari P., Zhang F.P., Poutanen M., Dufour J.F. (2016). Stress-activated miR-21/miR-21* in hepatocytes promotes lipid and glucose metabolic disorders associated with high-fat diet consumption. Gut.

[60] Francque S., Verrijken A., Caron S., Prawitt J., Paumelle R., Derudas B., Lefebvre P., Taskinen M.R., Van Hul W., Mertens I. (2015). PPARalpha gene expression correlates with severity and histological treatment response in patients with non-alcoholic steatohepatitis. J. Hepatol..

[61] Rodrigues P.M., Afonso M.B., Simao A.L., Carvalho C.C., Trindade A., Duarte A., Borralho P.M., Machado M.V., Cortez-Pinto H., Rodrigues C.M. (2017). miR-21 ablation and obeticholic acid ameliorate nonalcoholic steatohepatitis in mice. Cell Death Dis..

[62] Zhang X., Deng F., Zhang Y., Zhang X., Chen J., Jiang Y. (2021). PPARgamma attenuates hepatic inflammation and oxidative stress of non-alcoholic steatohepatitis via modulating the miR-21-5p/SFRP5 pathway. Mol. Med. Rep..

[63] Kalvari I., Nawrocki E.P., Ontiveros-Palacios N., Argasinska J., Lamkiewicz K., Marz M., Griffiths-Jones S., Toffano-Nioche C., Gautheret D., Weinberg Z. (2021). Rfam 14: Expanded coverage of metagenomic, viral and microRNA families. Nucleic Acids Res..

[64] Yang H., Li T.W., Peng J., Tang X., Ko K.S., Xia M., Aller M.A. (2011). A mouse model of cholestasis-associated cholangiocarcinoma and transcription factors involved in progression. Gastroenterology.

[65] Glaser S., Meng F., Han Y., Onori P., Chow B.K., Francis H., Venter J., McDaniel K., Marzioni M., Invernizzi P. (2014). Secretin stimulates biliary cell proliferation by regulating expression of microRNA 125b and microRNA let7a in mice. Gastroenterology.

[66] (2004). Gene [Internet]. Bethesda (MD): National Library of Medicine (US), National Center for Biotechnology Information. https://www.ncbi.nlm.nih.gov/gene/.

[67] Kozomara A., Birgaoanu M., Griffiths-Jones S. (2019). miRBase: From microRNA sequences to function. Nucleic Acids Res..

[68] Moslehi A., Hamidi-Zad Z. (2018). Role of SREBPs in Liver Diseases: A Mini-review. J. Clin. Transl. Hepatol..

[69] Zahm A.M., Wang A.W., Wang Y.J., Schug J., Wangensteen K.J., Kaestner K.H. (2020). A High-Content Screen Identifies MicroRNAs That Regulate Liver Repopulation After Injury in Mice. Gastroenterology.

[70] Chen S., Zhang Y., Ding X., Li W. (2022). Identification of lncRNA/circRNA-miRNA-mRNA ceRNA Network as Biomarkers for Hepatocellular Carcinoma. Front. Genet..

[71] Unfried J.P., Marin-Baquero M., Rivera-Calzada A., Razquin N., Martin-Cuevas E.M., de Braganca S., Aicart-Ramos C., McCoy C., Prats-Mari L., Arribas-Bosacoma R. (2021). Long Noncoding RNA NIHCOLE Promotes Ligation Efficiency of DNA Double-Strand Breaks in Hepatocellular Carcinoma. Cancer Res..

[72] Cheng T., Yao Y., Zhang S., Zhang X.N., Zhang A.H., Yang W., Hou C.Z. (2021). LINC00205, a YY1-modulated lncRNA, serves as a sponge for miR-26a-5p facilitating the proliferation of hepatocellular carcinoma cells by elevating CDK6. Eur. Rev. Med. Pharmacol. Sci..

[73] Meng H., Niu R., Huang C., Li J. (2022). Circular RNA as a Novel Biomarker and Therapeutic Target for HCC. Cells.

[74] Wu W., Ji P., Zhao F. (2020). CircAtlas: An integrated resource of one million highly accurate circular RNAs from 1070 vertebrate transcriptomes. Genome Biol..

[75] Yuan P., Qi X., Song A., Ma M., Zhang X., Lu C., Bian M., Lian N., He J., Zheng S. (2021). LncRNA MAYA promotes iron overload and hepatocyte senescence through inhibition of YAP in non-alcoholic fatty liver disease. J. Cell. Mol. Med..

[76] Juni R.P., t Hart K.C., Houtkooper R.H., Boon R.A. (2022). Long noncoding RNAs in cardiometabolic disorders. FEBS Lett..

[77] Jin Y., Wong Y.S., Goh B.K.P., Chan C.Y., Cheow P.C., Chow P.K.H., Lim T.K.H., Goh G.B.B., Krishnamoorthy T.L., Kumar R. (2019). Circulating microRNAs as Potential Diagnostic and Prognostic Biomarkers in Hepatocellular Carcinoma. Sci. Rep..

[78] Kim T.H., Lee Y., Lee Y.S., Gim J.A., Ko E., Yim S.Y., Jung Y.K., Kang S., Kim M.Y., Kim H. (2021). Circulating miRNA is a useful diagnostic biomarker for nonalcoholic steatohepatitis in nonalcoholic fatty liver disease. Sci. Rep..

[79] Chen M.J., Wang X.G., Sun Z.X., Liu X.C. (2019). Diagnostic value of LncRNA-MEG3 as a serum biomarker in patients with hepatitis B complicated with liver fibrosis. Eur. Rev. Med. Pharmacol. Sci..

[80] Zhang K., Han Y., Hu Z., Zhang Z., Shao S., Yao Q., Zheng L., Wang J., Han X., Zhang Y. (2019). SCARNA10, a nuclear-retained long non-coding RNA, promotes liver fibrosis and serves as a potential biomarker. Theranostics.

[81] Qiu L., Xu H., Ji M., Shang D., Lu Z., Wu Y., Tu Z., Liu H. (2019). Circular RNAs in hepatocellular carcinoma: Biomarkers, functions and mechanisms. Life Sci..

[82] Jacovetti C., Bayazit M.B., Regazzi R. (2021). Emerging Classes of Small Non-Coding RNAs With Potential Implications in Diabetes and Associated Metabolic Disorders. Front. Endocrinol..

